# Triple artemisinin-containing combination anti-malarial treatments should be implemented now to delay the emergence of resistance

**DOI:** 10.1186/s12936-019-2955-z

**Published:** 2019-10-11

**Authors:** Nicholas J. White

**Affiliations:** Mahidol Oxford Tropical Medicine Research Unit, Faculty of Tropical Medicine, Mahidol University, 420/6 Rajvithi Road, Bangkok, 10400 Thailand

## Abstract

Resistance threatens all our currently available anti-malarial drugs. Triple artemisinin-containing combination anti-malarial treatments (TACTs) combine an artemisinin derivative with two slowly eliminated partner drugs. TACTs are undergoing large-scale trials. If they prove safe, well-tolerated and efficacious then they should be deployed. This is in order to protect and extend the useful therapeutic life of the current generation of anti-malarial drugs, which are so essential for malaria control and elimination.

## Background

Short-term forecasting is improving all the time, but long-term predictions around complex systems (e.g. stock markets, fashion, weather) remain unreliable. Even when there is an overwhelming scientific consensus based upon ever increasing evidence (e.g. climate change, deforestation, evolution) firmly held opposing beliefs are maintained—often for religious or political, or even personal reasons. What on earth has this got to do with malaria? Being such an important disease, and such a complex problem, control and elimination of malaria have always courted lively debate and divergent opinions. Yet implementation of advances and innovations, changes in policies, and adoption of policy changes into practices are usually slow. Too slow. This is not a new problem. There is little pressure for responses to be rapid. Malaria affects particularly the young, the poor and the marginalized—people with little or no political power. Most of the money to control malaria comes from rich countries, which do not suffer from the disease. There is no strong patient lobby demanding better prevention and treatment of malaria. Those who make the big decisions are removed and cushioned from the harsh realities of relentless malaria and grinding poverty. There are endless meetings, but there seems never enough money for malaria control. In this context, suggesting that we spend more money, use more drugs and change policy when, for the most part, the currently recommended medicines are working well seems ridiculous. Is it?

Most people think that prevention is better than cure. In the public health sector investment in preventive measures, Governments and International Agencies do see the “value” in investing in vaccines, safe water, sanitation and vector control, and increasingly in public engagement and education. Curiously, they seem less willing to invest in ensuring the quality and longevity, or even the availability of existing medicines—despite the obvious health benefits. And now to the point! Resistance threatens all anti-infective drugs. There is belated recognition that more is needed to counter the threat of antimicrobial resistance. Recent initiatives have focussed on antibacterial resistance, but more is also needed to counter the threat of anti-malarial resistance. And it is not just about needing more money. We should do everything we can within reason to protect the anti-malarial drugs we have, as we have not been very good at inventing new ones. Despite investments of well over a billion dollars in anti-malarial drug development over the past two decades, the anti-malarial drugs that have made the major impact in this period are all more than 40 years old. The most important recent therapeutic advances in malaria have been the replacement of quinine by artesunate for the treatment of severe malaria (although sadly quinine is still often the only parenteral drug available for severely ill patients), and the replacement of failing anti-malarial monotherapies (chloroquine, amodiaquine, sulfadoxine–pyrimethamine and mefloquine) by artemisinin-based combinations for the treatment of uncomplicated falciparum malaria [1]. Together these medicines have saved millions of lives. But resistance to the artemisinins has developed. It has emerged first in South-East Asia, specifically in the eastern Greater Mekong subregion (GMS), where these drugs were widely available as monotherapies since the late 1980s. Ominously, this is exactly where chloroquine, and later sulfadoxine–pyrimethamine resistance were identified first. Drug resistant malaria parasites originating in the eastern GMS spread across India and then across Africa. Treatment failure rates rose, and then mortality rose, but international agencies supporting malaria control dragged their heels. For years, they were unwilling to endorse the new artemisinin-based combinations and continued to support increasingly ineffective anti-malarial drugs, while the death toll continued to rise [2]. Surely this should not be allowed to happen again?

Given how difficult it is to develop well tolerated, safe, simple to administer, affordable and highly effective anti-malarial drugs, and given the inevitability of resistance emerging and spreading, it seems wise to do everything that we can now to delay the emergence of resistance. The current approach is unsatisfactory; that is to wait until anti-malarial drug resistance has developed, wait longer until there are high treatment failure rates, and then to change policy. Even then there is usually a lengthy delay before the policy is actually translated into practice. During this protracted process the degree of resistance often worsens. This happened before with increasing levels of chloroquine resistance, then stepwise acquisition of point mutations in *Pfdhfr* and *Pfdhps* mediating resistance to antifols and sulfas, and most recently with piperaquine resistance in the eastern GMS where the acquisition (and spread) of mutations in *Pfcrt* has compounded piperaquine resistance [3]. At low levels of resistance drugs may still be useful in combinations, but this utility declines with increasing levels of resistance. It is accepted that combinations of anti-malarial drugs with different targets or resistance mechanisms should be deployed to delay the selection of de-novo resistance and thus prolong their effective therapeutic life [1]. This concept has been developed and strengthened over 70 years of anti-malarial drug resistance research. Wallace Peters, whose own research provided much of the evidence base, wrote over 30 years ago that “the only hope for anti-malarial drugs in the future is to apply them in appropriate, not randomly selected combinations” [4]. Implicit in this recommendation is that the components of the combination have to be effective, and provide mutual protection against resistance. The artemisinin-based combinations have done remarkably well, but they are imperfect combinations as the pharmacokinetic properties of the components are poorly matched. While the slowly eliminated partner (provided it still works) protects the artemisinin derivative by killing any spontaneously arising artemisinin-resistant mutant, the rapidly eliminated artemisinins are present for only 3 days (exposing two asexual cycles after starting the treatment) and so may leave up to 100,000 parasites in the third asexual cycle for the partner drug to remove alone [5]. In artemisinin-resistant infections this residuum increases by several orders of magnitude which explains why failure rates are higher and partner drug resistance is selected. Puzzlingly, this is sometimes referred to as “partial resistance” because susceptibility of mature asexual parasite stages is little affected (all anti-malarials show stage specificity in their effects), but the net result is a substantial reduction in efficacy—over one hundred times less parasite killing per asexual cycle in artemisinin resistant infections. Triple artemisinin-containing combination anti-malarial treatments (TACTs) combine an artemisinin derivative with two slowly eliminated partner drugs. After completion of the treatment regimen the rapidly eliminated artemisinin component provides no more parasite killing, but in TACTs there are now two more slowly eliminated drugs to deal together with the parasite residuum. This substantially increases the mutual protection by minimizing the exposure of the residual malaria parasites to a single drug. An alternative is to give two artemisinin-based combinations sequentially-currently a 6 days regimen, but longer courses of treatment risk reduced adherence, thereby confounding the original objective. It should be noted that combining three anti-malarials together is not new; the QAP regimen of quinine–atebrine (mepacrine, quinacrine)–plasmoquine (pamaquine) was used widely before and during the Second World War, and MSP (mefloquine–sulfadoxine–pyrimethamine) was promoted in Thailand in the 1980s.

TACTS are not quite ready. Their tolerability and safety are under assessment. Manufacturing, packaging, dose stratification, possible co-formulation etc. will need some investment—but nothing like the development of a completely new anti-malarial drug. The results so far with two potential candidate combinations (artemether–lumefantrine plus amodiaquine and dihydroartemisinin–piperaquine plus mefloquine) are reassuring [3] and large-scale studies will report in the near future. If TACTs do prove safe and well tolerated, then I think they should be deployed now, at least in areas currently affected or threatened by artemisinin resistance. The bigger question is whether they should be deployed in all malaria endemic areas. Today, we are heavily reliant on one artemisinin-based combination—artemether–lumefantrine. Over 500,000,000 treatment doses are taken each year. What will we turn to if we lose it? The emergence and spread of anti-malarial drug resistance in the Eastern GMS could be a harbinger of events elsewhere. In the face of worsening resistance Cambodia has switched from artesunate–mefloquine, to dihydroartemisinin–piperaquine, then back to artesunate–mefloquine and, with others in the region, has now been persuaded to adopt artesunate–pyronaridine. What is next? The new anti-malarial drugs in development will not be ready for general distribution within the next few years even if they do prove safe, and they are affordable. Should they be deployed like dominoes to fall sequentially, or should we take more active measures to protect them? The most pressing need now is to protect this generation of artemisinin-based combinations, that we rely upon so heavily to control malaria, to ensure they remain effective for as long as possible. TACTs would be a wise investment for the immediate future. Prevention is much better than cure.
